# MicroRNAs: The Missing Link in the Biology of Graft-Versus-Host Disease?

**DOI:** 10.3389/fimmu.2013.00420

**Published:** 2013-12-02

**Authors:** Sadaf Atarod, Anne Mary Dickinson

**Affiliations:** ^1^Haematological Sciences, Institute of Cellular Medicine, Newcastle University, Newcastle upon Tyne, UK

**Keywords:** biomarkers, graft-versus-host disease, microRNAs, bioinformatics, allogeneic hematopoietic stem cell transplantation

## Abstract

Graft-versus-host disease (GVHD) is still the major complication of allogeneic hematopoietic stem cell transplantation. Despite extensive studies in understanding the pathophysiology of GVHD, its pathogenesis remains unclear. Recently, important functions of microRNAs have been demonstrated in various autoimmune diseases and cancers such as psoriasis and lymphoma. This review highlights the need to investigate the role of microRNAs in GVHD and hypothesizes that microRNAs may be one of the missing links in our understanding of GVHD, with the potential for novel therapeutics.

## Introduction

Patients with hematopoietic malignancies such as acute myeloid leukemia, non-Hodgkins lymphoma, and Hodgkins lymphoma may be treated by allogeneic hematopoietic stem cell transplantation (allo-HSCT). However, there are severe complications associated with allo-HSCT such as disease relapse, graft-versus-host disease (GVHD), graft rejection, and infection mainly as a consequence of long term immuno-suppression (1). The extent of these complications can vary depending on the type of disease, stage of diagnosis, age of the transplant patient and whether the donor is Human Leukocyte Antigen (HLA) matched or mismatched to the patient.

Over the last 20 years, the field of allo-HSCT has advanced in numerous aspects. Various sources of hematopoietic stem cells (HSCs) have been utilized for allo-HSCT including; bone marrow, peripheral blood, and umbilical cord blood (UBC) (2–4). Likewise, there have been improvements in the HLA typing of patients and donors as well as earlier diagnosis of the underlying hematological disorders. Similarly, conditioning regimens have also changed from myeloablative to non-myeloablative incorporating less toxic regimens in many transplant centers across the globe, allowing more transplants in the older age group (45–65) and high risk categories. Patient care and drug regimens have improved with the introduction of new drugs such as calcineurin inhibitors for GVHD prophylaxis and imatinib mesylate for the treatment of chronic myelogenous leukemia. However, steroids still remain the first line therapy for GVHD treatment and steroid refractoriness can give rise to life threatening exacerbated GVHD and mortality (5).

Irrespective of all the improvements in the field of allo-HSCT, GVHD is still the most critical complication and the major cause of transplant related death. Manipulation of donor cells by subset depletion of specific T cell subsets have been used to facilitate engraftment and reduce GVHD (6, 7). The recognition of the host minor and major histocompatibility (MHC) antigens by the donor alloreactive T cells via host dendritic cells (DCs) results in the initiation of GVHD (8). In addition, there is some evidence that monocytes as precursors of conventional DCs may be more involved in GVHD initiation and propagation than donor DCs while plasmacytoid precursor DCs aid in regulating engraftment (9, 10).

The overall incidence of GVHD is approximately 50% in allo-HSCT patients (11). In addition, GVHD develops in two forms; acute and chronic. Acute GVHD (aGVHD) has been classically described as onset within the first 100 days of transplantation (12) [incidence of grade II–IV being 39% in sibling donors and 59% in Matched Unrelated Donor (MUD)] (13) where damage is observed in the skin, liver and gastrointestinal tract. Chronic GVHD (cGVHD) classically occurs after 100 days post-transplant (12) in 40% of HLA identical sibling transplants, 50% of HLA non-identical sibling transplants, and 70% of MUD transplants (14). Acute and cGVHD vary in pathophysiology, etiology, and response to treatment regimens (1). aGVHD is commonly characterized by a T helper 1 (Th1)-type cellular response (15), while cGVHD resembles autoimmune disorders (16). However, aGVHD and cGVHD have been observed to overlap, making a specific diagnosis between them difficult. This simultaneous presentation of acute and cGVHD after day 100 has been termed as “overlap syndrome” (17). The incidence of overlap syndrome appears to be increasingly common due to improved treatment methods (calcineurin inhibitors and reduced intensity conditioning regimens) (1).

In brief, aGVHD comprises of three main stages which are not completely separated from one another and can occur concurrently (8, 18). Initially, the patient’s tissues are damaged as a consequence of chemotherapy and conditioning regimens which results in the release of cytokines such as Interleukin (IL)-1, IL-6, Tumor Necrosis Factor (TNF), and bacterial products from the gut (lipopolysaccharides). Secondly, more host/donor antigen presenting cells (APCs) and donor T cells are recruited via these chemokines and activated. The final stage involves the “cytokine storm” whereby the host organs are attacked via cytotoxic T cells and the further release of cytokines such as Interferon Gamma (IFN-γ), TNF, IL-1, IL-2, IL-6, IL-21, IL-22, and IL-23 (18– 21). Several important cell types [donor T cells, macrophages, DCs, natural killer cells, regulatory T cells (Tregs), and B cells], chemokine receptors (CCR1, CCR2, CCR3, and CCR5), and cytokines (IFN-γ, IL-6, IL-8, IL-10, IL-18) have been identified as involved in the pathophysiology of GVHD (Table 1).

**Table 1:** **Potential biomarkers of GVHD [adopted from Ref. (22, 23)]**.

**BIOMARKERS OF aGVHD**
Interleukin-2 receptor α chain, CD25 (IL-2Rα)
Interleukin-6 (IL-6)
Interleukin-8 (IL-8)
Interleukin-10 (IL-10)
Interleukin-12 (IL-12)
Interleukin-18 (IL-18)
Chemokine (C–C motif) ligand 8 (CCL8)
Chemokine (C–X–C motif) ligand 10 (CXCL10)
Tumor necrosis factor α (TNFα)
Tumor necrosis factor receptor-1 (TNFR-1)
Hepatocyte growth factor (HGF)
Cytokeratin-18 fragments (KRT18)
Elafin (PI3)
Regenerating islet-derived 3 α (REG3α)
**BIOMARKER OF cGVHD**
B cell-activating factor (BAFF)

**Figure 1:**
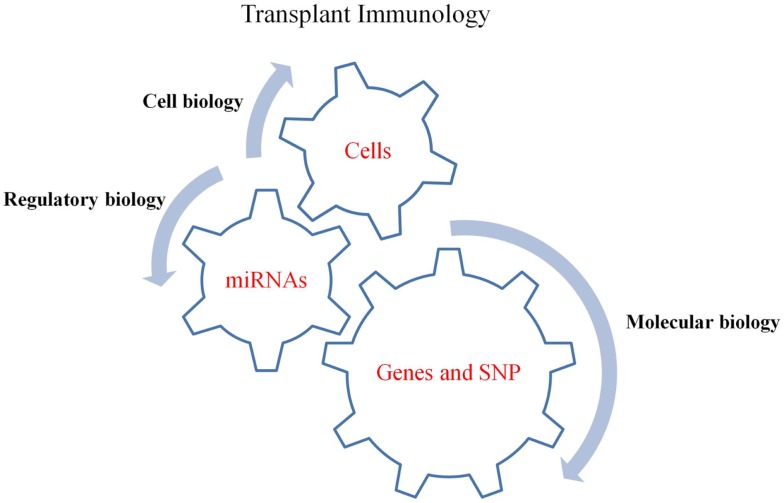
**The driving gears of GVHD**. MiRNAs regulate gene expressions, chemokine and cytokine secretions while their expression is simultaneously affected by SNPs in mRNA (gene) targets. Thus, understanding the involvement of miRNAs in regulating gene expression, which ultimately affect the function of cells, will aid in our better understanding of the regulatory mechanisms involved in GVHD pathogenesis.

The review by Paczesny lists the array of potential biomarkers that have been identified for the detection of GVHD in allo-HSCT patients (23). However, despite the tremendous advances in our knowledge of GVHD there are no precise clinical disease markers available in the clinic that can aid in early detection of GVHD and monitor its severity. At present, there is extensive knowledge on the cellular mechanism of GVHD but less is known about the molecular biology of the disease. The molecular studies carried out so far have focused on identifying Single Nucleotide Polymorphisms (SNPs) (24) and specific genes involved in the development of GVHD (25). However, there have been fewer studies focusing on the molecular regulation of GVHD (Figure 1). In a recent review, Paczesny et al. (26) has also highlighted the potential role of microRNAs (miRNAs/miRs) as one of the biomarkers of GVHD. Thus, the aim of this review is to describe the existing knowledge of miRNAs with regards to GVHD by addressing the various miRNA studies carried out in the cells involved at various stages of GVHD and also the recent GVHD specific miRNA investigations. It also aims to highlight the importance of miRNA investigations in the field of biomarker discovery and provide an insight into miRNA therapeutic applications, via their extensive regulatory roles.

## MicroRNAs

MicroRNAs are 19–22 nucleotide RNAs that are produced in eukaryotic cells (27). These short, single stranded RNAs have crucial regulatory roles by targeting messenger RNAs (mRNAs). As a result of the association between miRNA function and numerous diseases there has been an upsurge of databases that assist in miRNA target prediction, analysis of expression data, pathway involvement and interpretation of their roles in diseases. Examples of the miRNA databases include; DIANA LAB (28), microRNA.org (29), and TargetScan (30). Similarly, databases such as MAGIA (31) allow users to identify miRNA gene targets via their specific algorithms by submitting mRNA and miRNA expression results. MAGIA also enables users to perform metaanalysis on submission of expression results in unmatched samples (31).

## Biogenesis of microRNAs

**Figure 2:**
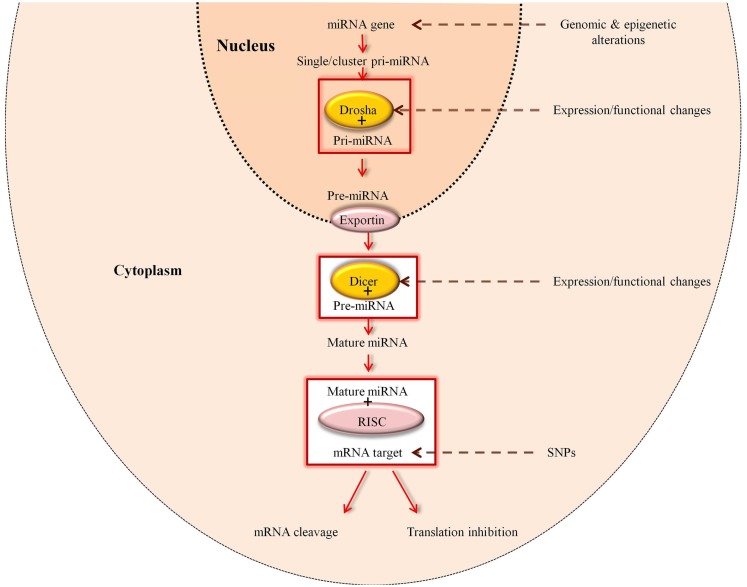
**Regulation of miRNAs**. Expression of miRNAs can be altered at various stages of its biogenesis by genomic (SNPs and mutations) and epigenetic alterations. Changes in the expression and function of Drosha and Dicer, which are part of the miRNA processing machinery, can also lead to the deregulation of mature miRNAs [adapted from Ref. (37)].

Primary miRNAs (pri-miRNAs) are transcribed from miRNA genes either as a cluster or as single molecules. Initially the pri-miRNA is cleaved by Drosha RNase-III, which is part of the microprocessor protein complex and resides in the nucleus (32). This cut results in a 5′ phosphate and a 3′ overhang with approximately 60–70 nucleotide stem loop structure referred to as precursor miRNA (pre-miRNA) (32–34). At this point, the pre-miRNA is translocated from the nucleus into the cytoplasm through Exportin-5 (35, 36) (Figure 2).

Dicer, the second enzyme involved in the maturation of the miRNA, is secreted in the cytoplasm (32) and has an affinity for the 5′ phosphate and the 3′ overhang of the pre-miRNA (27). The terminal base pairs of the pre-miRNA are cleaved by Dicer through a double-stranded cut at approximately two helical turns from the base of the pre-miRNA. At present it is hypothesized that Drosha cleaves at a specific sequence of the RNA, while Dicer randomly cuts any double-stranded RNA (38–41).

The miRNA duplex (miRNA-5p: miRNA-3p) comprises both strands of miRNA, of which commonly only the guide strand (miRNA-5p) is incorporated into the ribonucleoprotein complex. This contains a member of the Argonaute protein family and is termed the RNA Induced Silencing Complex (RISC) or the miRNA Induced Silencing Complex (miRISC) (42). Argonaute proteins act as the catalytic unit of the RISC complexes and are located in certain areas of the cytoplasm referred to as P-bodies (43). The P-body is the region where the majority of the mRNA degradation and miRNA activity occurs in the cytoplasm (43, 44). It was previously postulated that miRNA-3p was degraded, but more recent studies have revealed that both the guide (miRNA-5p) and passenger strand (miRNA-3p) are active and their roles depend on the specific condition in which they are expressed and processed (45, 46).

In addition, it has been recently shown that miRNAs can be processed by other non-canonical (non-classical) pathways (47) which either involve Dicer or act independently (48). To date, two other Dicer-dependent pathways have been described. The first pathway employs a spliceosome and then a debranching enzyme to generate the short-hairpin structure for processing by Dicer (47), while the second pathway uses unknown nucleases to generate the hair-pin structure which is later processed by Dicer. MiRNAs derived via the first pathway are termed as mitrons (49) while those originating from the second pathway are referred to as endogenous short-hairpin-derived miRNAs (50). In the Dicer-independent pathway, the pre-miRNA is cleaved by Argonaute 2 (Ago2) which results in the generation of mature miRNA.

MicroRNAs down regulate gene expression via the RISC by either mRNA cleavage or translational repression (27). The mechanism of regulation is dependent on the target mRNA. Messenger RNA cleavage is achieved when the incorporated miRNA within the RISC has extensive complementarity with the target mRNA and in cases of lesser complementarity (restricted to seed sequence), translational repression is employed. The seed sequence is the region of the miRNA which is approximately 6–8 nucleotides and is the site for mRNA target recognition (30). Upon cleavage the miRNA remains incorporated in the RISC to perform additional cleavages. The majority of miRNAs are tightly associated with RISC complexes and only 3% are present independently. It is due to this fact that miRNAs are stable and not degraded by nucleases in cells and thus, have an extremely long half-life (51–53).

## Regulation of microRNA and Its Processing Machinery

There are various factors that are involved in the biogenesis of miRNAs, including; RNase-III family proteins, double-stranded binding proteins and the export receptor. The RNAse-III family comprises of Drosha and Dicer which are both endonucleolytic enzymes (54). The double-stranded binding protein comprises the DiGeorge Syndrome Critical Region 8 (DGCR8), which is a well conserved motif with numerous functions. DGCR8 is partly involved in the processing of miRNA by Drosha and Dicer (54). The human export receptor (Expotin-5) consists of a nuclear transport domain (55) and mediates nuclear export of the double-stranded pre-miRNA into the cytoplasm. As evident from miRNA biogenesis, the miRNA processing machinery is important in the maturation of functional miRNAs. Thus, disruption of this machinery can result in deregulated miRNA expression. Studies have shown that the expression of skin-specific miRNAs such as miR-21, miR-203, and miR-125b are important in skin morphogenesis and deregulation can result in diseases such as psoriasis as reviewed by Banerjee et al. (56). Another recent investigation has also shown that low Dicer l levels in endometrial cancer cells can result in an over-expression of interferon beta (*IFN*-β). This is due to the accumulation of pre-miRNAs in the cytoplasm, which induce the transcription of interferon response genes such as Signal Transducers and Activators of Transcription family of transcription factors (*STAT1*) and Interferon-Induced Protein 44 (*IFI44*) (57).

In addition to defects in the miRNA processing machinery, mutations in the primary transcript such as those demonstrated in miR-15a and miR-16-1 in chronic lymphocytic leukemia (58) can also impact on miRNA expression levels. Since miRNA–mRNA interaction is based on the complementarity of the seed sequence with the mRNA, it has been shown that SNPs in the miRNA genes play a role in the processing and function of the miRNA (59). Moreover, the presence of SNPs in the miRNA target can also affect the function of miRNA (59). Chromosomal abnormalities, epigenetic factors and transcriptional factors can also lead to miRNA deregulation (60) (Figure 2). Thus, individual alteration in the processing of miRNAs and their seed sequence can deregulate miRNA expression and in turn, disrupt the homeostasis of cells, and cause variation in gene and protein expression patterns between diseased and normal tissues. Consequently, investigation of the components of the miRNA processing machinery as well as mature miRNA expression levels with regard to GVHD may help to explain the differential gene expression patterns observed in GVHD patients compared to patients without GVHD or healthy controls.

## MicroRNAs as Biomarkers of Diseases

Recent biomarker literature has focused on the numerous characteristics of miRNAs as potential and specific biomarkers of various diseases, including rheumatoid arthritis (61) and cancer (62). Studies have shown that around 50% of all genes are regulated by miRNAs, thereby further emphasizing the importance of understanding their involvement in disease (63). Hence, miRNAs are classed as one of the major and most abundant group of translational regulators (64). According to miRbase version 18, there are 1921 miRNAs in humans (65). Since most of the miRNAs in cells and tissues have been identified, it is now possible to perform expression studies in GVHD samples in order to develop a miRNA signature for the disease.

As mentioned earlier, miRNAs are not only abundant, but are also highly stable due to their resistance to nucleases (66). This fact enables expression studies using formalin fixed-paraffin-embedded (FFPE) samples (67) and salivary samples possible (68). Since, most research institutions possess archives of patient samples; a substantial number of relevant patient materials (serum, plasma, FFPE, urine) are available for biomarker discovery investigations. Also, studies have shown that there is a correlation between serum and biopsy miRNA profiles in a number of cancer studies as reviewed by Alevizos and Illei (61). Mitchell et al. (69) have demonstrated that circulating miRNAs in plasma and serum are stable and that their measurements correlate with each other. This finding strengthens the hypothesis that tumor-derived miRNAs are translocated into the blood and hence, measurement of plasma or serum derived miRNAs may serve as cancer biomarkers (69). The availability of potential circulatory diagnostic miRNAs (61) could reduce the need for invasive methods such as skin biopsies for the diagnosis of GVHD in allo-HSCT patients and aid in disease monitoring. Moreover, miRNAs are expressed under certain conditions which can be representative of the physiological and pathological state of the disease such as rheumatic diseases (61). MicroRNAs have been shown to exhibit functionally unique cell or organ expression patterns (27). For instance, miR-1 is a heart specific miRNA (70) while miR-122 is mainly expressed in the liver (71). Since, miRNA expression patterns are disease specific, distinguishing between normal, inflamed and damaged organs is possible (72). MicroRNA patterns already established in systemic lupus erythematosus and other systemic autoimmune diseases can be useful in understanding the pathogenesis of cGVHD (73).

Although a single prognostic marker may be clinically useful, a series of validated markers which can provide, in additional to clinical factors, information regarding survival, disease progression, and patient’s response to treatment would be advantageous. Thus, identification of a signature miRNA profile for GVHD could serve both as a prognostic biomarker of the disease as well as aid in further understanding the complexity of the disease biology. Specific biomarkers can also aid in the development of new therapies and more effective drugs for treatment of GVHD.

## MicroRNAs in Skin

Since skin is one of the first target organs of GVHD, the role of skin-specific miRNAs in GVHD skin biopsies is an interesting area of investigation. A number of highly expressed miRNAs in the epidermis and hair follicle have already been discovered to be essential for the normal development of the skin (for example: miR-199 family, miR-205, miR-27b, miR-203, miR-125b, miR-16, miR-126, miR-143, miR-21) (56, 74). Skin-specific miRNAs such as miR-203 are related to skin morphogenesis (75, 76). Indeed, expression studies have shown the effect of miRNAs in malignant skin conditions such as melanoma, Kaposi’s sarcoma (77), and autoimmune diseases such as psoriasis (78). For instance in psoriasis, miR-146a, miR-203, and miR-21 are up-regulated while miR-125b is down-regulated (79). Up-regulation of TNF-α is observed when miR-125b is expressed at low levels (76) and similarly, expression of miR-21 is shown to be elevated by pro-inflammatory cytokines such as IL-6 (80). MiR-21 has also been shown to function as an oncogene in various cancer tissues (81, 82). Although miR-21 has been shown to be associated with Tregs (see below) there is a lack of evidence to suggest that the impact of miR-21 in skin tissue is primarily via Tregs and no other cell type.

## MicroRNAs in Immune Cells

Thymus-derived natural Treg cells and the peripherally stimulated Tregs are well characterized T lymphocytes and their function in GVHD has been highlighted through various studies. Natural Tregs and peripherally induced Tregs are distinguished via the expression of the fork head-winged helix transcription factor (FOXP3) and the α-chain of the IL-2 receptor (CD25) (83). A study performed *ex vivo* in humans by Rouas et al. (83) has identified five main miRNAs (miR-21, miR-31, miR-125a, miR-181c, and miR-374) specific to non-activated natural Tregs (83). Natural Tregs affect both the innate and adaptive immune system (84). Moreover, the same group demonstrated the direct negative regulation of miR-31 through targeting of *FOXP3* mRNA and positive indirect regulation of *FOXP3* by miR-21 (83).

Furthermore, Allantaz et al. (85) have shown the existence of cell-specific miRNAs in the whole blood of normal individuals. Initially, they investigated miRNA expression in nine different types of immune cells comprising of, B cells, neutrophils, eosinophils, NK cells, CD4 T cells, CD8 T cells, myeloid DCs, plasmacytoid DCs, and monocytes (85). Four miRNAs were characterized as cell specific (miR-378, miR-31, miR-143, and miR-935) while nine miRNAs were common in two to three other cell types (miR-362-5p, miR-532-5p, miR-500^∗^, miR-663, miR-125a-5p, miR-150, miR-223, and miR-652) (85) (Table 2). The group also investigated mRNA expression of the miRNA targets in the same samples to validate whether the cell-specific miRNAs regulated their predicted mRNA targets, as identified via miRNA target prediction databases. MiRs-143, -125, -500, -150, -652, and -223 were all found to regulate their mRNA target transcripts (85). These investigations reiterate the critical regulatory roles of miRNAs in immune cells and provide a valuable starting point for miRNA studies in GVHD.

**Table 2:** **List of cell-specific miRNAs in whole blood of normal individuals [adopted from Ref. (85)]**.

miRNAs	Cell type
miR-378	Monocytes
miR-31	T cells
miR-935	Eosinophils
miR-143	Neutrophils
miR-362-5p	Monocytes, pDCs
miR-532-5p	
miR-500*	
miR-663	B cells, NK cells
miR-125a-5p	T cells, neutrophils
miR-150	B cells, T cells, NK cells
miR-223	Monocytes, eosinophils, neutrophils
miR-652	

pDCs, plasmacytoid dendritic cells; NK cells, natural killer cells.

## MicroRNAs in GVHD

Recently, a panel comprising of four miRNAs (miR-423, miR-199-3p, miR-93*, and miR-377) has been shown to be over-expressed 16 days pre-clinical diagnosis, in the plasma of aGVHD patients when compared to the non-GVHD patient cohort (86). All the up-regulated miRNAs have critical functions in the regulation of inflammation, cell proliferation, apoptosis and autophagy. Xiao et al. (86) hypothesize that plasma identified miRNAs may have significant functions in “donor T cells attacking process” and play a potential role in injury-mediated responses in aGVHD target organs. In addition, miR-100 (87), miR-34a (88), and miR-155 (89) have also been implicated as playing a potentially significant role in GVHD. MiR-100 was shown to be up-regulated in the gut of mice without GVHD, thereby preventing neovascularization in the tissue (87) and demonstrating a possible protective role of miR-100 in GVHD (87). Likewise, miR-34a expression was studied in the gut of pre- and post-transplanted Fanconi Anemia patients with aGVHD (88). MiR-34a was shown to be up-regulated in the gut of the transplanted Fanconi Anemia patients with grades II-IV aGVHD in comparison to patients with grades 0-I aGVHD and pre-transplant biopsies (88). DNA repair mechanisms are disrupted in Fanconi Anemia patients and this result in the activation of p53 pathways, ultimately leading to apoptosis (90). Thus, the investigators assessed the number of apoptotic cells in relation to the levels of miR-34a and *TP53* (88). They found that miR-34a levels in the gut correlated with the number of apoptotic cells and not *TP53* levels (88). Therefore, they hypothesized that it was the elevation of miR-34a in the epithelial gut cells which was responsible for the damage observed in the gut tissues rather than *TP53* expression (88). Similarly, regulatory function of miR-155, which is required for the normal function of B and T lymphocytes in humans, has been demonstrated in an aGVHD study (89). This investigation showed an up-regulation of miR-155 in the gut of aGVHD patients, while expression was absent in the gut of normal volunteers (89). A clinical trial to further establish the significance of miR-155 in aGVHD is ongoing at present (ClinicalTrials.gov identifier: NCT01521039). Thus, miRNAs evidently not only play a role in the manifestation of GVHD but could potentially be used as biomarkers of the disease because of their highly specialized roles. Moreover, Schulte et al. (91) have demonstrated that miRNAs have specialized functions and a hierarchy in regulating inflammation. In their investigation, miR-155 is significantly involved in the regulation of inflammation only when the regulatory limit of miR-146a has been exceeded (91). Their study highlights the combinatorial function of miRNAs in regulating inflammation and also shows that investigating several miRNAs important in a disease-type can provide a more informative outlook on the pathophysiology of the disease.

**Figure 3:**
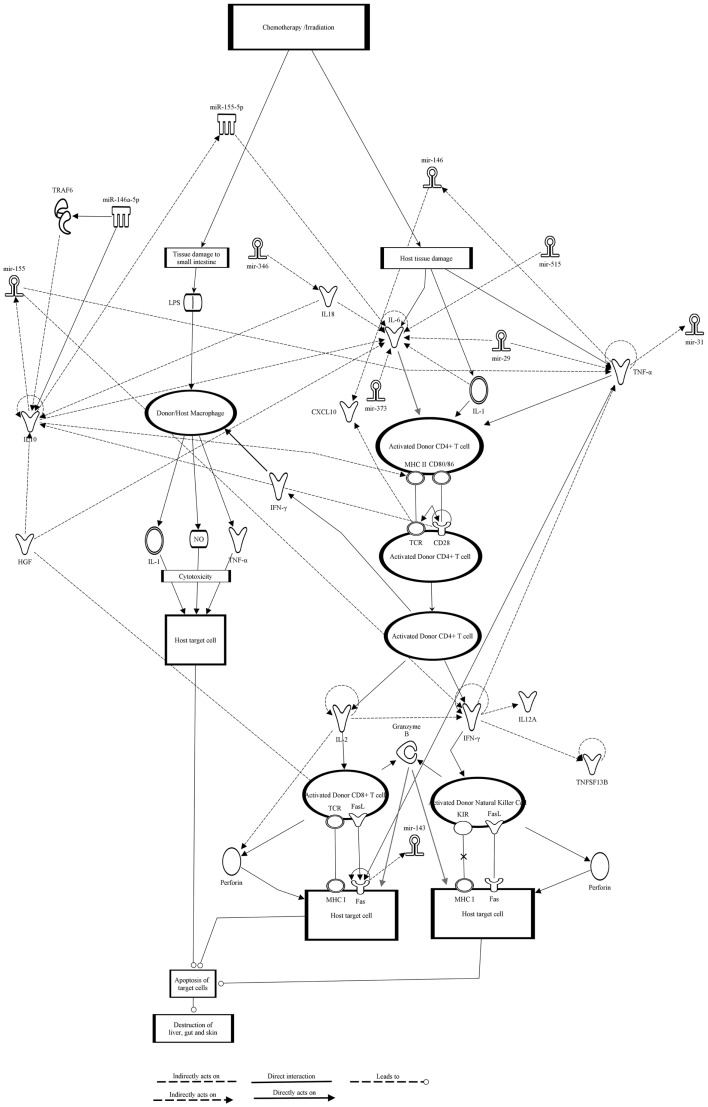
**Graft-versus-host disease signaling pathway and miRNA interactions obtained using IPA**. Classical GVHD signaling pathway was selected from the IPA knowledge base and eight miRNAs were identified to interact with the various components of the pathway. Chemokines and genes are represented as nodes of various shapes and the biological relationship and interactions between them are represented as a line. Direct experimentally proven interactions are represented with a solid line, while indirect interactions are shown as a dashed line. All the interactions are supported by at least one reference from either the literature or from the information available in the Ingenuity Pathways Knowledge Base. The direction of the interaction is indicated with the arrow head. miR-146a-5p and miR-155-5p are mature miRNAs while miR-515, miR-346, miR-143, miR-373, miR-31, and miR-29 could be primary, precursor or even mature forms of miRNAs.

In addition, using Ingenuity Pathway Analysis (IPA) we generated the GVHD signaling pathway and identified potential miRNA interactions (92). We used the canonical GVHD signaling pathway present in the Ingenuity Knowledge Base of IPA and added further GVHD related chemokine receptors and cytokines. Eight miRNAs (miR-146a, miR-155, miR-515, miR-346, miR-143, miR-373, miR-31, and miR-29) were identified to impact different molecules in this GVHD signaling pathway (Figure 3). All the miRNAs identified have significant roles in numerous diseases and a summary is shown in Table 3. Due to the exponential growth of literature on miRNAs it is out of the limits of this review to completely list all the diseases for which miRNAs have been implicated. As mentioned earlier, miR-146a and miR-155 have immuno-regulatory roles in inflammation and numerous diseases, thus it is expected that their levels may be deregulated in GVHD. Interestingly, miR-515 is part of a cluster of miRNAs transcribed from a gene locus on chromosome 19 (65) (miR-515-2, miR-512-1, miR-1323, miR-498, miR-520e, miR-519e, miR-520f, miR-519d, and miR-1283-1). Similarly, the miR-29 family comprises of four members (miR-29a, miR-29b-1, miR-29b-2, and miR-29c).

**Table 3:** **miRNAs identified via Ingenuity Pathway Knowledge base to be implicated in the GVHD signaling pathway**.

miRNA	Disease
miR-146a	↑Psoriasis (78, 79), ↓myelodysplastic syndrome (5q deletion) (93) ↓systemic lupus erythematosus (94)
miR-155	↑aGVHD (89), ↓systemic sclerosis (95), ↓oral tumors (96)
miR-515	↑Oral tumors (96)
miR-346	↓Lupus nephritis (97), regulates TNF-α protein in rheumatoid arthritis (98)
miR-143	↓Myelodysplastic syndrome (5q deletion) (99)
miR-373	↓Childhood B cell precursor acute lymphoblastic leukemia (100)
miR-31	↑Colorectal cancer (101)
miR-29	↓Hepatocellular carcinoma (102)

## MicroRNAs in Umbilical Cord Blood

Weitzel et al. (103) have demonstrated miR-184 as a regulator of nuclear factor of activated T cells-1 (NFAT1) protein expression in UCB CD4^+^ T cells. Expression of miR-184 inhibits NFAT1, which results in a reduced inflammatory response. The lower expression of NFAT1 in UCB CD4^+^ T cells in comparison to adult blood is one of the main differences between UCB-derived CD4^+^ T cells and those derived from adult blood. This study has shown that over-expression of miR-184 in UCB CD4^+^ T cells may be a reason for the decreased incidence of GVHD in UCB grafts compared to adult hematopoietic stem cell transplants (103). In addition, Charrier et al. (104) have also demonstrated that UCBs express significantly higher levels of miR-146a and miR-155 compared to adult blood. Previous studies have shown that miR-146a (78, 79) and miR-155 (89, 105) are associated with immune regulation. Thus, they hypothesize that the lower incidence of GVHD which occurs when UCB is used instead of adult blood is due to the up-regulated expression of immune-regulatory miRNAs (miR-146a and miR-155), resulting in the down regulation of proteins (toll-like receptor 9, myeloid differentiation primary response 88, IL-1 receptor-associated kinase 1, interferon regulatory factor 7) in the toll-like receptor 9 signaling pathway (104). This post-transcriptional regulation leads to a decrease in the interferon-α mediated response, which dampens down the inflammatory reaction in UCBs as opposed to adult blood (104). In addition, it has also been shown that miR-155 is over expressed in UCB-derived CD14^+^ cells and not in the adult peripheral blood CD14^+^ cells when stimulated with either IFN-γ or lipopolysaccharide (106). This may be reflective of the biological response in GVHD or infection. However, in the same study miR-146a was under expressed in UCB-derived CD14^+^ cells after stimulation with IFN-γ. The controversy regarding miR-146a expression may be reflective of study populations investigated. Indeed, Charrier et al. (104) have looked at whole UCB and adult blood rather than an isolated CD14^+^ subset as used by Takahashi et al. (106). Merkerova et al. (107) compared UCB and bone marrow miRNA signatures in cell lineages CD34^+^ cells, T cells, monocytes, and granulocytes and found specific miRNA expression patterns indicating differences in regulation of the cells within bone marrow and UCB.

## MicroRNAs in Graft Rejection

Investigations in renal allograft rejection have shown the existence of 17 specific miRNAs involved in the acute rejection process (let-7c, miR-10a, miR-10b, miR-125a, miR-200a, miR-30a-3p, miR-30b, miR-30c, miR-30e-3p, miR-32, miR-142-5p, miR-142-3p, miR-223, miR-155, miR-146b, miR-146a, and miR-342) (108). This study also highlights the application of miRNAs as biomarkers for disease and in particular for transplantation. The significance of miRNAs in graft failure has also been determined in liver, lung, and bowel solid organ transplants as reviewed by Sarma et al. (109). Furthermore in hematopoietic stem cell transplantation some residual host T cells or natural killer cells can reject incoming grafts (110) and high levels of CD34^+^ cells can improve engraftment (111). A study in the role of miRNAs in these situations would aid in the understanding of hematopoiesis, engraftment and rejection settings.

## MicroRNAs in Therapeutics

**Figure 4:**
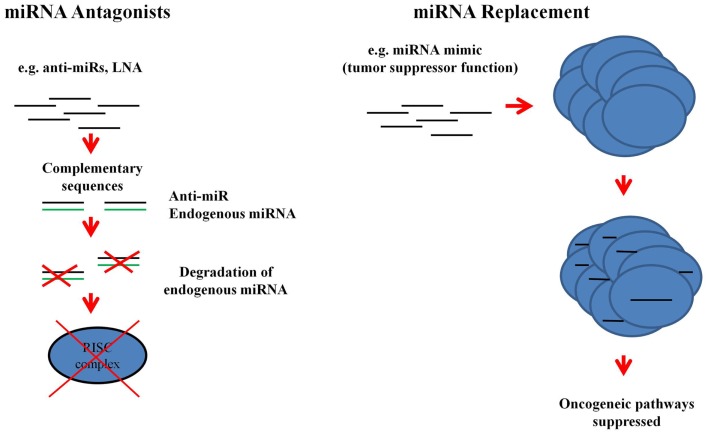
**Therapeutic applications of miRNAs**. MiRNA antagonists, which are modified oligonucleotides with complementary sequences to the endogenous miRNAs, can be used to degrade over-expressed miRNAs. Loss of function of the endogenous miRNA prevents it being processed by RISC. MiRNA mimics are also oligonucleotides, but they replace the lost function due to the disease state of the cell [adopted from Ref. (117)].

Recently, the potential of miRNAs in the development of new therapies has been investigated. There are two models via which miRNAs may be used for the treatment of diseases (Figure 4). Firstly, miRNA antagonists can be used to dampen up-regulated endogenous miRNA expression in diseased tissues. For instance, miR-122, which is a liver specific miRNA, has a crucial function in the replication of hepatitis C virus (HCV) and is up-regulated in HCV-positive patients (60, 112, 113). Miravirsen (anti-miR-122) which consists of modified locked nucleic acids (LNA) is now in a phase II clinical trial (ClinicalTrials.gov Identifier: NCT01727934). Miravirsen efficacy, activity, and safety has been shown in a multi center Phase I (ClinicalTrials.gov Identifiers: NCT00688012, NCT00979927) and Phase IIa (ClinicalTrials.gov Identifier: NCT01200420) clinical trial. Miravirsen sequesters miR-122 and inhibits it from binding to the HCV genome thereby preventing its RNA multiplication (114). Thus, miravirsen is a potential new therapy for the treatment of HCV-positive patients. Secondly, miRNA mimics, such as miR-34a (115) and let-7 (116), can be used to restore the function of miRNAs which are lost in the diseased cells (117). Unlike gene therapy which can be difficult to achieve because of the need to introduce large plasmids into the target tissue, miRNA mimics can be delivered using silencing RNA technology. The fact that miRNA mimics are small and representative of the endogenous miRNA sequences means that mRNA target specificity is increased and thus off-target effects (targeting many mRNAs) are less problematic. MiRNA mimics target the same mRNAs as the endogenous miRNA population which is lost due to the disease (117). With the array of technologies that are constantly being developed and made available in the miRNA field, normalization of deregulated miRNAs is feasible. Similarly, more effective therapeutic options such as the anti-miR in HCV can be developed once the signature miRNAs in GVHD have been discovered.

## Future Outlook

Although miRNA studies in the field of GVHD are in their infancy, recent investigations have demonstrated the tremendous potential for these small regulatory molecules as diagnostic, prognostic, and therapeutic markers. Clinical applications exploiting our knowledge of miRNA function may play a crucial role in the future of GVHD management and treatment.

Our own studies are currently focusing on the use of clinical skin biopsies for assessment of miRNA in GVHD diagnosis and the use of serum, plasma, and urine for investigating the potential of miRNAs in monitoring GVHD in response to therapy. Once miRNA profiles are established, functional studies involving silencing of miRNA will assess the potential of specific miRNAs as therapeutic targets.

## Conflict of Interest Statement

The authors declare that the research was conducted in the absence of any commercial or financial relationships that could be construed as a potential conflict of interest.

## References

[1] Welniak LA Blazar BR Murphy WJ Immunobiology of allogeneic hematopoietic stem cell transplantation. Annu Rev Immunol (2007) 25:139–70 10.1146/annurev.immunol.25.022106.141606 17129175

[2] Schmitz N Dreger P Suttorp M Rohwedder EB Haferlach T Loffler H Primary transplantation of allogeneic peripheral blood progenitor cells mobilized by filgrastim (granulocyte colony-stimulating factor). Blood (1995) 85:1666–72 7534141

[3] Wagner JE Kernan NA Steinbuch M Broxmeyer HE Gluckman E Allogeneic sibling umbilical-cord-blood transplantation in children with malignant and non-malignant disease. Lancet (1995) 346:214–9 10.1016/S0140-6736(95)91268-1 7616801

[4] Barker JN Wagner JE Umbilical-cord blood transplantation for the treatment of cancer. Nat Rev Cancer (2003) 3:526–32 10.1038/nrc1125 12835672

[5] Westin JR Saliba RM De Lima M Alousi A Hosing C Qazilbash MH Steroid-refractory acute GVHD: predictors and outcomes. Adv Hematol (2011) 2011:8 10.1155/2011/601953 22110505 PMC3216266

[6] Martin PJ Donor CD8 cells prevent allogeneic marrow graft rejection in mice: potential implications for marrow transplantation in humans. J Exp Med (1993) 178:703–12 10.1084/jem.178.2.703 8101864 PMC2191137

[7] Gandy KL Domen J Aguila H Weissman IL CD8+TCR+ and CD8+TCR- cells in whole bone marrow facilitate the engraftment of hematopoietic stem cells across allogeneic barriers. Immunity (1999) 11:579–90 10.1016/S1074-7613(00)80133-8 10591183

[8] Ferrara JL Levine JE Reddy P Holler E Graft-versus-host disease. Lancet (2009) 373:1550–61 10.1016/S0140-6736(09)60237-3 19282026 PMC2735047

[9] Fugier-Vivier IJ Rezzoug F Huang Y Graul-Layman AJ Schanie CL Xu H Plasmacytoid precursor dendritic cells facilitate allogeneic hematopoietic stem cell engraftment. J Exp Med (2005) 201:373–83 10.1084/jem.20041399 15699072 PMC2213023

[10] Bonnefoy F Perruche S Couturier M Sedrati A Sun Y Tiberghien P Plasmacytoid dendritic cells play a major role in apoptotic leukocyte-induced immune modulation. J Immunol (2011) 186:5696–705 10.4049/jimmunol.1001523 21460208

[11] Pavletic SZ Fowler DH Are we making progress in GVHD prophylaxis and treatment? Hematology Am Soc Hematol Educ Program (2012) 2012:251–64 10.1182/asheducation-2012.1.251 23233589

[12] Shlomchik WD Graft-versus-host disease. Nat Rev Immunol (2007) 7:340–52 10.1038/nri2000 17438575

[13] Jagasia M Arora M Flowers ME Chao NJ McCarthy PL Cutler CS Risk factors for acute GVHD and survival after hematopoietic cell transplantation. Blood (2012) 119:296–307 10.1182/blood-2011-06-364265 22010102 PMC3251233

[14] Apperley J Masszi T Graft-versus-host disease. 6 ed In: Apperley J Carreras E Gluckman E Masszi T, editors. EBMT-ESH Handbook on Haemopoietic Stem Cell Transplantation. Genoa: Forum service editor (2012). p. 216–33

[15] Mohty M Blaise D Faucher C Vey N Bouabdallah R Stoppa AM Inflammatory cytokines and acute graft-versus-host disease after reduced-intensity conditioning allogeneic stem cell transplantation. Blood (2005) 106:4407–11 10.1182/blood-2005-07-2919 16141347

[16] Graze PR Gale RP Chronic graft versus host disease: a syndrome of disordered immunity. Am J Med (1979) 66:611–20 10.1016/0002-9343(79)91171-9 35001

[17] Filipovich AH Weisdorf D Pavletic S Socie G Wingard JR Lee SJ National institutes of health consensus development project on criteria for clinical trials in chronic graft-versus-host disease: I. Diagnosis and Staging Working Group Report. Biol Blood Marrow Transplant (2005) 11:945–56 10.1016/j.bbmt.2005.09.004 16338616

[18] Blazar BR Murphy WJ Abedi M Advances in graft-versus-host disease biology and therapy. Nat Rev Immunol (2012) 12:443–58 10.1038/nri3212 22576252 PMC3552454

[19] Ferrara JL Cytokine dysregulation as a mechanism of graft versus host disease. Curr Opin Immunol (1993) 5:794–9 10.1016/0952-7915(93)90139-J 8240742

[20] Krenger W Hill GR Ferrara JL Cytokine cascades in acute graft-versus-host disease. Transplantation (1997) 64:553–8 10.1097/00007890-199708270-00001 9293864

[21] Couturier M Lamarthee B Arbez J Renauld JC Bossard C Malard F IL-22 deficiency in donor T cells attenuates murine acute graft-versus-host disease mortality while sparing the graft-versus-leukemia effect. Leukemia (2013) 27:1527–37 10.1038/leu.2013.39 23399894

[22] Levine JE Paczesny S Sarantopoulos S Clinical applications for biomarkers of acute and chronic graft-versus-host disease. Biol Blood Marrow Transplant (2012) 18:S116–24 10.1016/j.bbmt.2011.10.019 22226094 PMC3282925

[23] Paczesny S Discovery and validation of graft-versus-host disease biomarkers. Blood (2012) 121(4):585–94 10.1182/blood-2012-08-355990 23165480 PMC3557644

[24] Dickinson AM Holler E Polymorphisms of cytokine and innate immunity genes and GVHD. Best Pract Res Clin Haematol (2008) 21:149–64 10.1016/j.beha.2008.03.004 18503983

[25] Baron C Somogyi R Greller LD Rineau V Wilkinson P Cho CR Prediction of graft-versus-host disease in humans by donor gene-expression profiling. PLoS Med (2007) 4:e23 10.1371/journal.pmed.0040023 17378698 PMC1796639

[26] Paczesny S Raiker N Brooks S Mumaw C Graft-versus-host disease biomarkers: omics and personalized medicine. Int J Hematol (2013) 98(3):275–92 10.1007/s12185-013-1406-9 23959582 PMC4005044

[27] Bartel DP MicroRNAs: genomics, biogenesis, mechanism, and function. Cell (2004) 116:281–97 10.1016/S0092-8674(04)00045-5 14744438

[28] Alexiou P Maragkakis M Papadopoulos GL Simmosis VA Zhang L Hatzigeorgiou AG The DIANA-mirExTra web server: from gene expression data to microRNA function. PLoS One (2010) 5:e9171 10.1371/journal.pone.0009171 20161787 PMC2820085

[29] Betel D Wilson M Gabow A Marks DS Sander C The microRNA.org resource: targets and expression. Nucleic Acids Res (2008) 36:D149–53 10.1093/nar/gkm995 18158296 PMC2238905

[30] Lewis BP Burge CB Bartel DP Conserved seed pairing, often flanked by adenosines, indicates that thousands of human genes are microRNA targets. Cell (2005) 120:15–20 10.1016/j.cell.2004.12.035 15652477

[31] Sales G Coppe A Bisognin A Biasiolo M Bortoluzzi S Romualdi C MAGIA, a web-based tool for miRNA and genes integrated analysis. Nucleic Acids Res (2010) 38:W352–9 10.1093/nar/gkq423 20484379 PMC2896126

[32] Lee Y Ahn C Han J Choi H Kim J Yim J The nuclear RNase III Drosha initiates microRNA processing. Nature (2003) 425:415–9 10.1038/nature01957 14508493

[33] Lee Y Jeon K Lee JT Kim S Kim VN MicroRNA maturation: stepwise processing and subcellular localization. EMBO J (2002) 21:4663–70 10.1093/emboj/cdf476 12198168 PMC126204

[34] Zeng Y Yi R Cullen BR MicroRNAs and small interfering RNAs can inhibit mRNA expression by similar mechanisms. Proc Natl Acad Sci U S A (2003) 100:9779–84 10.1073/pnas.1630797100 12902540 PMC187842

[35] Yi R Qin Y Macara IG Cullen BR Exportin-5 mediates the nuclear export of pre-microRNAs and short hairpin RNAs. Genes Dev (2003) 17:3011–6 10.1101/gad.1158803 14681208 PMC305252

[36] Lund E Guttinger S Calado A Dahlberg JE Kutay U Nuclear export of microRNA precursors. Science (2004) 303:95–8 10.1126/science.1090599 14631048

[37] Iorio MV Croce CM MicroRNAs in cancer: small molecules with a huge impact. J Clin Oncol (2009) 27:5848–56 10.1200/JCO.2009.24.0317 19884536 PMC2793003

[38] Zamore PD Tuschl T Sharp PA Bartel DP RNAi: double-stranded RNA directs the ATP-dependent cleavage of mRNA at 21 to 23 nucleotide intervals. Cell (2000) 101:25–33 10.1016/S0092-8674(00)80620-0 10778853

[39] Bernstein E Caudy AA Hammond SM Hannon GJ Role for a bidentate ribonuclease in the initiation step of RNA interference. Nature (2001) 409:363–6 10.1038/35053110 11201747

[40] Elbashir SM Lendeckel W Tuschl T RNA interference is mediated by 21- and 22-nucleotide RNAs. Genes Dev (2001) 15:188–200 10.1101/gad.862301 11157775 PMC312613

[41] Zhang H Kolb FA Brondani V Billy E Filipowicz W Human Dicer preferentially cleaves dsRNAs at their termini without a requirement for ATP. EMBO J (2002) 21:5875–85 10.1093/emboj/cdf582 12411505 PMC131079

[42] Sonkoly E Pivarcsi A Advances in microRNAs: implications for immunity and inflammatory diseases. J Cell Mol Med (2009) 13:24–38 10.1111/j.1582-4934.2008.00534.x 19175698 PMC3823034

[43] Filipowicz W Bhattacharyya SN Sonenberg N Mechanisms of post-transcriptional regulation by microRNAs: are the answers in sight? Nat Rev Genet (2008) 9:102–14 10.1038/nrg2290 18197166

[44] Williams AE Functional aspects of animal microRNAs. Cell Mol Life Sci (2008) 65:545–62 10.1007/s00018-007-7355-9 17965831 PMC11131689

[45] Ro S Park C Young D Sanders KM Yan W Tissue-dependent paired expression of miRNAs. Nucleic Acids Res (2007) 35:5944–53 10.1093/nar/gkm641 17726050 PMC2034466

[46] Okamura K Phillips MD Tyler DM Duan H Chou YT Lai EC The regulatory activity of microRNA* species has substantial influence on microRNA and 3’ UTR evolution. Nat Struct Mol Biol (2008) 15:354–63 10.1038/nsmb.1409 18376413 PMC2698667

[47] Gangaraju VK Lin H MicroRNAs: key regulators of stem cells. Nat Rev Mol Cell Biol (2009) 10:116–25 10.1038/nrm2621 19165214 PMC4118578

[48] Yang JS Maurin T Lai EC Functional parameters of Dicer-independent microRNA biogenesis. RNA (2012) 18:945–57 10.1261/rna.032938.112 22461413 PMC3334703

[49] Okamura K Hagen JW Duan H Tyler DM Lai EC The mirtron pathway generates microRNA-class regulatory RNAs in *Drosophila*. Cell (2007) 130:89–100 10.1016/j.cell.2007.06.028 17599402 PMC2729315

[50] Babiarz JE Ruby JG Wang Y Bartel DP Blelloch R Mouse ES cells express endogenous shRNAs, siRNAs, and other Microprocessor-independent, Dicer-dependent small RNAs. Genes Dev (2008) 22:2773–85 10.1101/gad.1705308 18923076 PMC2569885

[51] Liu J Carmell MA Rivas FV Marsden CG Thomson JM Song JJ Argonaute2 is the catalytic engine of mammalian RNAi. Science (2004) 305:1437–41 10.1126/science.1102513 15284456

[52] Martinez J Tuschl T RISC is a 5’ phosphomonoester-producing RNA endonuclease. Genes Dev (2004) 18:975–80 10.1101/gad.1187904 15105377 PMC406288

[53] Tang F Hajkova P O’Carroll D Lee C Tarakhovsky A Lao K MicroRNAs are tightly associated with RNA-induced gene silencing complexes in vivo. Biochem Biophys Res Commun (2008) 372:24–9 10.1016/j.bbrc.2008.04.137 18474225 PMC7612963

[54] Kim VN MicroRNA biogenesis: coordinated cropping and dicing. Nat Rev Mol Cell Biol (2005) 6:376–85 10.1038/nrm1644 15852042

[55] Nakielny S Dreyfuss G Transport of proteins and RNAs in and out of the nucleus. Cell (1999) 99:677–90 10.1016/S0092-8674(00)81666-9 10619422

[56] Banerjee J Chan YC Sen CK MicroRNAs in skin and wound healing. Physiol Genomics (2011) 43:543–56 10.1152/physiolgenomics.00157.2010 20959495 PMC3110888

[57] Chiappinelli KB Haynes BC Brent MR Goodfellow PJ Reduced DICER1 elicits an interferon response in endometrial cancer cells. Mol Cancer Res (2012) 10:316–25 10.1158/1541-7786.MCR-11-0520 22252463 PMC3307918

[58] Raveche ES Salerno E Scaglione BJ Manohar V Abbasi F Lin YC Abnormal microRNA-16 locus with synteny to human 13q14 linked to CLL in NZB mice. Blood (2007) 109:5079–86 10.1182/blood-2007-02-071225 17351108 PMC1890829

[59] Sun G Yan J Noltner K Feng J Li H Sarkis DA SNPs in human miRNA genes affect biogenesis and function. RNA (2009) 15:1640–51 10.1261/rna.1560209 19617315 PMC2743066

[60] Iorio M Casalini P Piovan C Braccioli L Tagliabue E Current and future developments in cancer therapy research: miRNAs as new promising targets or tools. In: Bologna M, editor. Biotargets of Cancer in Current Clinical Practice. Milan: Humana Press (2012). p. 517–46 10.1007/978-1-61779-615-9_19

[61] Alevizos I Illei GG MicroRNAs as biomarkers in rheumatic diseases. Nat Rev Rheumatol (2010) 6:391–8 10.1038/nrrheum.2010.81 20517293 PMC3041596

[62] Lawrie CH Soneji S Marafioti T Cooper CD Palazzo S Paterson JC MicroRNA expression distinguishes between germinal center B cell-like and activated B cell-like subtypes of diffuse large B cell lymphoma. Int J Cancer (2007) 121:1156–61 10.1002/ijc.22800 17487835

[63] Bentwich I Prediction and validation of microRNAs and their targets. FEBS Lett (2005) 579:5904–10 10.1016/j.febslet.2005.09.040 16214134

[64] Lewis BP Shih IH Jones-Rhoades MW Bartel DP Burge CB Prediction of mammalian microRNA targets. Cell (2003) 115:787–98 10.1016/S0092-8674(03)01018-3 14697198

[65] Griffiths-Jones S miRBase: the microRNA sequence database. Methods Mol Biol (2006) 342:129–38 10.1385/1-59745-123-1:129 16957372

[66] Lau NC Lim LP Weinstein EG Bartel DP An abundant class of tiny RNAs with probable regulatory roles in *Caenorhabditis elegans*. Science (2001) 294:858–62 10.1126/science.1065062 11679671

[67] Hasemeier B Christgen M Kreipe H Lehmann U Reliable microRNA profiling in routinely processed formalin-fixed paraffin-embedded breast cancer specimens using fluorescence labelled bead technology. BMC Biotechnol (2008) 8:90 10.1186/1472-6750-8-90 19038028 PMC2605753

[68] Park NJ Zhou H Elashoff D Henson BS Kastratovic DA Abemayor E Salivary microRNA: discovery, characterization, and clinical utility for oral cancer detection. Clin Cancer Res (2009) 15:5473–7 10.1158/1078-0432.CCR-09-0736 19706812 PMC2752355

[69] Mitchell PS Parkin RK Kroh EM Fritz BR Wyman SK Pogosova-Agadjanyan EL Circulating microRNAs as stable blood-based markers for cancer detection. Proc Natl Acad Sci U S A (2008) 105:10513–8 10.1073/pnas.0804549105 18663219 PMC2492472

[70] Lee RC Ambros V An extensive class of small RNAs in *Caenorhabditis elegans*. Science (2001) 294:862–4 10.1126/science.1065329 11679672

[71] Lagos-Quintana M Rauhut R Yalcin A Meyer J Lendeckel W Tuschl T Identification of tissue-specific microRNAs from mouse. Curr Biol (2002) 12:735–9 10.1016/S0960-9822(02)00809-6 12007417

[72] Babak T Zhang W Morris Q Blencowe BJ Hughes TR Probing microRNAs with microarrays: tissue specificity and functional inference. RNA (2004) 10:1813–9 10.1261/rna.7119904 15496526 PMC1370668

[73] Shen N Liang D Tang Y De Vries N Tak PP MicroRNAs – novel regulators of systemic lupus erythematosus pathogenesis. Nat Rev Rheumatol (2012) 8:701–9 10.1038/nrrheum.2012.142 23070646

[74] Yi R O’Carroll D Pasolli HA Zhang Z Dietrich FS Tarakhovsky A Morphogenesis in skin is governed by discrete sets of differentially expressed microRNAs. Nat Genet (2006) 38:356–62 10.1038/ng1744 16462742

[75] Yi R Poy MN Stoffel M Fuchs E A skin microRNA promotes differentiation by repressing ‘stemness’. Nature (2008) 452:225–9 10.1038/nature06642 18311128 PMC4346711

[76] Sand M Gambichler T Sand D Skrygan M Altmeyer P Bechara FG MicroRNAs and the skin: tiny players in the body’s largest organ. J Dermatol Sci (2009) 53:169–75 10.1016/j.jdermsci.2008.10.004 19058951

[77] McClure LV Sullivan CS Kaposi’s sarcoma herpes virus taps into a host microRNA regulatory network. Cell Host Microbe (2008) 3:1–3 10.1016/j.chom.2007.12.002 18191785

[78] Sonkoly E Wei T Janson PC Saaf A Lundeberg L Tengvall-Linder M MicroRNAs: novel regulators involved in the pathogenesis of psoriasis? PLoS One (2007) 2:e610 10.1371/journal.pone.0000610 17622355 PMC1905940

[79] Sonkoly E Stahle M Pivarcsi A MicroRNAs: novel regulators in skin inflammation. Clin Exp Dermatol (2008) 33:312–5 10.1111/j.1365-2230.2008.02804.x 18419608

[80] Loffler D Brocke-Heidrich K Pfeifer G Stocsits C Hackermuller J Kretzschmar AK Interleukin-6 dependent survival of multiple myeloma cells involves the Stat3-mediated induction of microRNA-21 through a highly conserved enhancer. Blood (2007) 110:1330–3 10.1182/blood-2007-03-081133 17496199

[81] Liu L Wang YL Wang JF [Differential expression of miR-21, miR-126, miR-143, miR-373 in normal cervical tissue, cervical cancer tissue and Hela cell]. Sichuan Da Xue Xue Bao Yi Xue Ban (2012) 43:536–9 22997891

[82] Yang M Shen H Qiu C Ni Y Wang L Dong W High expression of miR-21 and miR-155 predicts recurrence and unfavourable survival in non-small cell lung cancer. Eur J Cancer (2012) 49(3):604–15 10.1016/j.ejca.2012.09.031 23099007

[83] Rouas R Fayyad-Kazan H El Zein N Lewalle P Rothe F Simion A Human natural Treg microRNA signature: role of microRNA-31 and microRNA-21 in FOXP3 expression. Eur J Immunol (2009) 39:1608–18 10.1002/eji.200838509 19408243

[84] Sakaguchi S Naturally arising CD4+ regulatory t cells for immunologic self-tolerance and negative control of immune responses. Annu Rev Immunol (2004) 22:531–62 10.1146/annurev.immunol.21.120601.141122 15032588

[85] Allantaz F Cheng DT Bergauer T Ravindran P Rossier MF Ebeling M Expression profiling of human immune cell subsets identifies miRNA-mRNA regulatory relationships correlated with cell type specific expression. PLoS One (2012) 7:e29979 10.1371/journal.pone.0029979 22276136 PMC3262799

[86] Xiao B Wang Y Li W Baker M Guo J Corbet K Plasma microRNA signature as a non-invasive biomarker for acute graft-versus-host disease. Blood (2013) 122(19):3365–75 10.1182/blood-2013-06-510586 24041574 PMC3821726

[87] Leonhardt F Grundmann S Behe M Bluhm F Dumont RA Braun F Inflammatory neovascularization during graft-versus-host disease is regulated by alphav integrin and miR-100. Blood (2013) 121(17):3307–18 10.1182/blood-2012-07-442665 23327924

[88] Wang L Romero M Ratajczak P Leboeuf C Belhadj S Peffault de Latour R Increased apoptosis is linked to severe acute GVHD in patients with Fanconi anemia. Bone Marrow Transplant (2012) 48(6):849–53 10.1038/bmt.2012.237 23222379

[89] Ranganathan P Heaphy CE Costinean S Stauffer N Na C Hamadani M Regulation of acute graft-versus-host disease by microRNA-155. Blood (2012) 119:4786–97 10.1182/blood-2011-10-387522 22408260 PMC3367879

[90] Freie B Li X Ciccone SL Nawa K Cooper S Vogelweid C Fanconi anemia type C and p53 cooperate in apoptosis and tumorigenesis. Blood (2003) 102:4146–52 10.1182/blood-2003-03-0971 12855557

[91] Schulte LN Westermann AJ Vogel J Differential activation and functional specialization of miR-146 and miR-155 in innate immune sensing. Nucleic Acids Res (2013) 41:542–53 10.1093/nar/gks1030 23143100 PMC3592429

[92] The graft-versus-host disease signalling pathway and microRNA interations were generated through the use of Ingenuity Pathway Analysis Ingenuity^®^ Systems. Available from: www.ingenuity.com [Online] (2013).

[93] Votavova H Grmanova M Dostalova Merkerova M Belickova M Vasikova A Neuwirtova R Differential expression of microRNAs in CD34+ cells of 5q- syndrome. J Hematol Oncol (2011) 4:1 10.1186/1756-8722-4-1 21211043 PMC3024999

[94] Tang Y Luo X Cui H Ni X Yuan M Guo Y MicroRNA-146a contributes to abnormal activation of the type I interferon pathway in human lupus by targeting the key signaling proteins. Arthritis Rheum (2009) 60:1065–75 10.1002/art.24436 19333922

[95] Nakashima T Jinnin M Yamane K Honda N Kajihara I Makino T Impaired IL-17 signaling pathway contributes to the increased collagen expression in scleroderma fibroblasts. J Immunol (2012) 188:3573–83 10.4049/jimmunol.1100591 22403442

[96] Scapoli L Palmieri A Lo Muzio L Pezzetti F Rubini C Girardi A MicroRNA expression profiling of oral carcinoma identifies new markers of tumor progression. Int J Immunopathol Pharmacol (2010) 23:1229–34 21244772 10.1177/039463201002300427

[97] Dai Y Sui W Lan H Yan Q Huang H Huang Y Comprehensive analysis of microRNA expression patterns in renal biopsies of lupus nephritis patients. Rheumatol Int (2009) 29:749–54 10.1007/s00296-008-0758-6 18998140

[98] Semaan N Frenzel L Alsaleh G Suffert G Gottenberg JE Sibilia J miR-346 controls release of TNF-alpha protein and stability of its mRNA in rheumatoid arthritis via tristetraprolin stabilization. PLoS One (2011) 6:e19827 10.1371/journal.pone.0019827 21611196 PMC3096642

[99] Dostalova Merkerova M Krejcik Z Votavova H Belickova M Vasikova A Cermak J Distinctive microRNA expression profiles in CD34+ bone marrow cells from patients with myelodysplastic syndrome. Eur J Hum Genet (2011) 19:313–9 10.1038/ejhg.2010.209 21150891 PMC3061996

[100] Ju X Li D Shi Q Hou H Sun N Shen B Differential microRNA expression in childhood B-cell precursor acute lymphoblastic leukemia. Pediatr Hematol Oncol (2009) 26:1–10 10.1080/08880010802378338 19206004

[101] Bandres E Cubedo E Agirre X Malumbres R Zarate R Ramirez N Identification by Real-time PCR of 13 mature microRNAs differentially expressed in colorectal cancer and non-tumoral tissues. Mol Cancer (2006) 5:29 10.1186/1476-4598-5-29 16854228 PMC1550420

[102] Xiong Y Fang JH Yun JP Yang J Zhang Y Jia WH Effects of microRNA-29 on apoptosis, tumorigenicity, and prognosis of hepatocellular carcinoma. Hepatology (2010) 51:836–45 10.1002/hep.23380 20041405

[103] Weitzel RP Lesniewski ML Haviernik P Kadereit S Leahy P Greco NJ microRNA 184 regulates expression of NFAT1 in umbilical cord blood CD4+ T cells. Blood (2009) 113:6648–57 10.1182/blood-2008-09-181156 19286996 PMC2710921

[104] Charrier E Cordeiro P Cordeau M Dardari R Michaud A Harnois M Post-transcriptional down-regulation of toll-like receptor signaling pathway in umbilical cord blood plasmacytoid dendritic cells. Cell Immunol (2012) 276:114–21 10.1016/j.cellimm.2012.04.010 22578600

[105] Zhong H Xu L Zhong JH Xiao F Liu Q Huang HH Clinical and prognostic significance of miR-155 and miR-146a expression levels in formalin-fixed/paraffin-embedded tissue of patients with diffuse large B-cell lymphoma. Exp Ther Med (2012) 3:763–70 10.3892/etm.2012.502 22969965 PMC3438582

[106] Takahashi N Nakaoka T Yamashita N Profiling of immune-related microRNA expression in human cord blood and adult peripheral blood cells upon proinflammatory stimulation. Eur J Haematol (2012) 88:31–8 10.1111/j.1600-0609.2011.01707.x 21913990

[107] Merkerova M Vasikova A Belickova M Bruchova H MicroRNA expression profiles in umbilical cord blood cell lineages. Stem Cells Dev (2010) 19(1): 17–26 10.1089/scd.2009.0071 19435428

[108] Anglicheau D Sharma VK Ding R Hummel A Snopkowski C Dadhania D MicroRNA expression profiles predictive of human renal allograft status. Proc Natl Acad Sci U S A (2009) 106:5330–5 10.1073/pnas.0813121106 19289845 PMC2663998

[109] Sarma NJ Tiriveedhi V Ramachandran S Crippin J Chapman W Mohanakumar T Modulation of immune responses following solid organ transplantation by microRNA. Exp Mol Pathol (2012) 93:378–85 10.1016/j.yexmp.2012.09.020 23036474 PMC3518685

[110] Martin PJ Winning the battle of graft versus host. Nat Med (2000) 6:18–9 10.1038/72230 10613813

[111] Weaver CH Hazelton B Birch R Palmer P Allen C Schwartzberg L An analysis of engraftment kinetics as a function of the CD34 content of peripheral blood progenitor cell collections in 692 patients after the administration of myeloablative chemotherapy. Blood (1995) 86:3961–9 7579367

[112] Haussecker D Kay MA miR-122 continues to blaze the trail for microRNA therapeutics. Mol Ther (2010) 18:240–2 10.1038/mt.2009.313 20125164 PMC2839286

[113] Lanford RE Hildebrandt-Eriksen ES Petri A Persson R Lindow M Munk ME Therapeutic silencing of microRNA-122 in primates with chronic hepatitis C virus infection. Science (2010) 327:198–201 10.1126/science.1178178 19965718 PMC3436126

[114] Janssen HL Reesink HW Lawitz EJ Zeuzem S Rodriguez-Torres M Patel K Treatment of HCV infection by targeting microRNA. N Engl J Med (2013) 368:1685–94 10.1056/NEJMoa1209026 23534542

[115] Wiggins JF Ruffino L Kelnar K Omotola M Patrawala L Brown D Development of a lung cancer therapeutic based on the tumor suppressor microRNA-34. Cancer Res (2010) 70:5923–30 10.1158/0008-5472.CAN-10-0655 20570894 PMC2913706

[116] Trang P Medina PP Wiggins JF Ruffino L Kelnar K Omotola M Regression of murine lung tumors by the let-7 microRNA. Oncogene (2010) 29:1580–7 10.1038/onc.2009.445 19966857 PMC2841713

[117] Bader AG Brown D Winkler M The promise of microRNA replacement therapy. Cancer Res (2010) 70:7027–30 10.1158/0008-5472.CAN-10-2010 20807816 PMC2940943

